# Dynamical analysis of a stochastic rumor-spreading model with Holling II functional response function and time delay

**DOI:** 10.1186/s13662-020-03096-9

**Published:** 2020-11-23

**Authors:** Liang’an Huo, Xiaomin Chen

**Affiliations:** Business School, University of Shanghai for Science and Technology, Shanghai, 200093 China

**Keywords:** Rumor spreading, Holling II functional response function, Time delay, Stochastic process, Asymptotic behavior

## Abstract

With the rapid development of information society, rumor plays an increasingly crucial part in social communication, and its spreading has a significant impact on human life. In this paper, a stochastic rumor-spreading model with Holling II functional response function considering the existence of time delay and the disturbance of white noise is proposed. Firstly, the existence of a unique global positive solution of the model is studied. Then the asymptotic behavior of the global solution around the rumor-free and rumor-local equilibrium nodes of the deterministic system is discussed. Finally, through some numerical results, the validity and availability of theoretical analysis is verified powerfully, and it shows that some factors such as the transmission rate, the intensity of white noise, and the time delay have significant relationship with the dynamical behavior of rumor spreading.

## Introduction

Rumor spreading has had a significant impact on human life, especially in the modern era of Internet, there is no geographical limit to rumor spreading, which brings much wider and faster spreading. To some extent, these rumors may cause individual panic, even lead to tremendous social unrest. Recently, during the outbreak of COVID-19, various negative rumors spread widely, which undoubtedly destroyed the psychosocial environment and threatened the national security [1–6]. Therefore, there is great significance of studying the mechanism of rumor spreading to grasp its dynamical properties.

There is no doubt that the problems of rumor spreading have been paid widely attention to by researchers. A classical DK model of rumor spreading, was proposed by Daley and Kendall [7] in the 1960s. Subsequently, the MT model proposed by Maki and Thompson made relevant corrections on the basis of the DK model [8]. However, both the DK and MK models didn’t take the topological characteristics of social networks into account, which can’t apply to the complex social network. In terms of this, Zanette first established a rumor spreading model on a small-world network based on the theory of complex networks [9]. In addition, Moreno et al. built a rumor-spreading model on a scale-free network and compared the numerical simulation results with random analysis results, which revealed that the rumor spreading has a great relationship with the topology structure and parameter setting of the network [10]. Besides, some microscopic mechanisms also are involved in rumor-spreading models, such as forgetting or memory mechanisms. In this respect, the others made enormous contribution, which revealed the influence of these mechanisms on rumor spreading [11, 12]. The above studies place emphasis on the final size of the rumor, while less concerning the dynamical process of rumor spreading [13–15]. Recently, Huo and Song established a 4D dynamic model of rumor spreading and concluded that the dynamic behavior is related to the rumor-spreading rate [16]. Then, they studied the dynamics of a novel spreading model considering the nonlinear incidence rate and the influence of the propagation environment [17]. Zhu investigated how the time delay factor affects the dynamics around the equilibrium points [18]. Jia et al. considered a rumor-spreading model with stochastic noise [19]. In general, existing studies mostly investigate the dynamics of rumor spreading from a certain respect [20–22].

In regard of the previous rumor-spreading models, they are mostly established with a bilinear incidence rate. However, in fact, compared with the bilinear incidence rate, a nonlinear incidence rate is more logical when considering individual psychological factors [23, 24]. Specially, Holling proposed the Holling II functional response function which is used to demonstrate the interaction between two communities in later models [25]. Besides, time delay exists in numerous dynamical systems [26–29], and especially there is no exception in a rumor-spreading system which covers people’s subjective initiative. It is not immediate that the rumor will spread in the crowd, requiring time for a person to think or recognize and commit to spread, that is to say, time delay indeed exists in the process of rumor spreading. And it is well known that the occurrence of time delay not only influences the dynamics of the model, but also changes the stability of the system. Furthermore, rumor is largely disturbed by the external environmental factors [30–32], especially in public emergencies. Take COVID-19 for example, when a rumor such as ‘Dual yellow Oral Liquid can effectively inhibit novel coronavirus’ spread widely, Dual yellow Oral Liquid was quickly sold out. When the government and media started refuting rumors and explaining the truth, the rumor spreading was suppressed, and at the same time people’s cognition changed accordingly and people were guided to behave correctly. Thus, with the changes in the environment, the spreading of rumor may vary accordingly. To investigate the dynamical properties of rumor spreading more precisely, it is essential to take stochastic disturbance into account [19, 22].

Above all, a rumor-spreading model only considering unilateral factor is far from adequate, both the time delay of thinking and the external disturbance from media or government, etc., may exert great influence on the dynamic behavior of this model. In this paper, we establish a rumor spreading model which takes Holling II functional response function, time delay, and stochastic disturbance into consideration simultaneously, it could be further accord with practical situation and grasp the dynamic characteristic of rumor spreading more precisely. Then we principally discuss the dynamic properties of the modified rumor-spreading model around the rumor-free and rumor-local equilibrium. The framework of the paper is summarized as follows. In Sect. 2, we propose a stochastic SIR rumor-spreading model with Holling II functional response function and time delay. In Sect. 3, we demonstrate the existence and uniqueness of the global positive solution. In Sect. 4, we investigate the asymptotic behavior around the rumor-free equilibrium. Similarly, the asymptotic behavior around the rumor-local equilibrium is studied in Sect. 5. In Sect. 6, we carry out some numerical simulations to verify the validity and availability of the above theorems. Finally, we summarize the main results and come to some conclusions in Sect. 7.

## A stochastic rumor-spreading model with Holling II functional response function and time delay

In this section, we first present a deterministic SIR rumor-spreading model with Holling II functional response function considering the existence of time delay. Primarily on the basis of the classical rumor-spreading model, the total population $N(t)$ is divided into three categories: susceptible $S(t)$, infected $I(t)$, and stifler $R(t)$, where $S(t)$ represents the members who haven’t heard the rumor at time *t*, $I(t)$ denotes the infected individuals who believe in and spread the rumor actively at time *t*, and $R(t)$ represents the individuals who have been removed from the infected population and stop spreading at time *t*. The rumor is propagated through the population by pairwise contacts between spreaders and others in the population, following the law of mass action [8]. Any spreader involved in a pairwise meeting attempts to “infect” the other individual with the rumor. In the case this other individual is ignorant, he/she becomes a spreader. In the other two cases, either one or both of those involved in the meeting learn that the rumor is “known” and decide not to tell the rumor anymore, thereby turning into stiflers. Besides, in the process of rumor spreading, the law of spreading between the susceptible and infected individuals matches the Holling II functional response function. On the one hand, individuals may believe in and spread the rumor in the essence of identification ability to rumors; on the other hand, the government may strengthen refuting rumors as the intensity of rumor spreading gradually increases. In general, the nonlinear incidence rate is much more realistic than the bilinear incidence rate. Thus, in order to consider the individual psychological factors and in accordance with reality, we introduced Holling II functional response function $\frac{\beta I(t)}{1 + \alpha I(t)}$ into this rumor-spreading model, where *β* means the transmission rate, *α* is used to measure the extent to which the infected individuals $I(t)$ inhibit the transmission. Furthermore, it takes time for individuals to react and identify when contacting with a rumor, that is to say, the existence of thinking or identifying time further conforms to the rumor in a real world. Thus, for the sake of predicting the dynamic behavior of rumor spreading more accurately, we consider a time delay in our model, where *τ* is defined as the time delay which denotes an identification period after receiving the rumor, $e^{ - \mu \tau }$ is the removal rate during the identification or thinking period, $\frac{1}{1 + \alpha I(t - \tau )}$ is used to measure the psychological influence of the progressive increase of the infected individuals $I(t)$ among the susceptible individuals $S(t)$. The model is given by

1 $$ \textstyle\begin{cases} \frac{dS(t)}{dt} = (1 - p)b - \frac{\beta S(t)I(t)}{1 + \alpha I(t)} - \mu S(t), \\ \frac{dI(t)}{dt} = e^{ - \mu \tau } \frac{\beta S(t - \tau )I(t - \tau )}{1 + \alpha I(t - \tau )} - \eta I(t)(I(t) + R(t)) - \mu I(t), \\ \frac{dR(t)}{dt} = pb + \eta I(t)(I(t) + R(t)) - \mu R(t), \end{cases} $$

where the parameter *p* denotes immunization rate of the new members, *b* denotes a constant input of new individuals into a given community, *μ* denotes the natural removal rate, *η* denotes immunization rate transforming from the infected into immune individuals, where we suppose that the transformation has two approaches: on the one hand, when the infected individuals $I(t)$ contact with the immune individuals $R(t)$, $I(t)$ turn into $R(t)$ with a certain probability because $R(t)$ have lost interest in the matter; on the other hand, when an infected individual $I(t)$ meets another infected individual $I(t)$, they both lose interest in spreading the rumor with a certain probability. All parameter values are set as positive constants.

In model (1), the basic reproduction number is

2 $$ R_{0} = e^{ - \mu \tau } \frac{\beta (1 - p)b}{\eta pb + \mu ^{2}}. $$

It is a threshold value that tells whether the rumor vanishes or not. If $R_{0} < 1$, the model (1) only has a rumor-free equilibrium $P_{0} = (S_{0},0,R_{0})$ and it has global asymptotic stability in the invariant set Γ, where $S_{0} = \frac{(1 - p)b}{\mu }$, $R_{0} = \frac{pb}{\mu }$, $\Gamma = \{ (S,I,R) \in \mathbb{R}_{+}^{3}:S + e^{\mu \tau } I + R \le \frac{b}{\mu } \} $. It means that the rumor will vanish. Conversely, if $R_{0} > 1$, the model (1) has a unique rumor-local equilibrium $P^{*} = (S^{*},I^{*},R^{*})$, where $I^{*} = \frac{e^{ - \mu \tau } (1 - p)b\beta \mu - pb\eta \mu - \mu ^{3}}{e^{ - \mu \tau } (1 - p)b\beta \eta + (pb\eta + \mu ^{2})(\beta + \mu \alpha )}$ and it has global asymptotic stability in the invariant set Γ under certain conditions, which means the rumor will propagate and be popular.

However, to some extent, rumor spreading is inevitably subject to the influence by various inside variation and outside environment disturbance. Especially in emergencies, as the event itself develops and varies, the government may establish a mechanism to refute rumors or the media may make a strategy adjustment to report, which is different from the classical rumor-spreading model. Obviously, stochastic models may forecast the future dynamic behavior more precisely. On the basis of the deterministic model considering the Holling II functional response function and time delay, we take the stochastic disturbance into consideration so that we can better grasp the dynamic properties. Here, we add the white noise based on the model (1), which makes the system more realistic and precise than the deterministic model. The stochastic SIR rumor-spreading model with time delay is given by

3 $$ \textstyle\begin{cases} dS(t) = [(1 - p)b - \frac{\beta S(t)I(t)}{1 + \alpha I(t)} - \mu S(t)]\,dt + \sigma _{1} S(t)\,dB_{1}(t), \\ dI(t) = [e^{ - \mu \tau } \frac{\beta S(t - \tau )I(t - \tau )}{1 + \alpha I(t - \tau )} - \eta I(t)(I(t) + R(t)) - \mu I(t)]\,dt + \sigma _{2} I(t)\,dB_{2}(t), \\ dR(t) = [pb + \eta I(t)(I(t) + R(t)) - \mu R(t)]\,dt + \sigma _{3} R(t)\,dB_{3}(t), \end{cases} $$

where $B_{1}(t)$, $B_{2}(t)$, and $B_{3}(t)$ are real-valued Brownian motions which are defined on the complete probability space $(\Omega , F, \{ F_{t}\}_{t \ge 0}, \mathrm{P})$with a filtration $\{ F_{t}\}_{t \ge 0}$ satisfying the usual conditions (i.e., it is right continuous and increasing while $F_{0}$ contains all P-null sets), and $\sigma _{i}$ ($i = 1,2,3$) are the intensities of white noise.

## Existence and uniqueness of the global positive solution

To study the dynamical properties of the stochastic system, the first concern is whether there’s a positive and global solution. As we all know, when the coefficients of the equation satisfy either the local Lipschitz condition or the linear growth condition (mentioned in [33]), we can ensure that the solution of a stochastic differential equation is positive and global. Nevertheless, the model (3) doesn’t meet the later condition, that is to say, the solution of the model (3) might explode in a finite time.

In the following part, we will demonstrate that the model (3) has global positive solutions in accordance with the Lyapunov analysis method (mentioned in [34]).

### Theorem 3.1

*For any given initial value* $(S(\theta ),I(\theta ),R(\theta )) \in \mathbb{R}_{3}^{ +}$, $\theta \in [ - \tau ,0]$, *the model* (3) *has a unique global solution* $(S(t), I(t), R(t))$ *for* $t \ge - \tau $ *and the solution will remain in* $\mathbb{R}_{ +}^{3}$ *with probability one*, *in other words*, $(S(t),I(t),R(t)) \in \mathbb{R}_{ +}^{3}$ *for all* $t \ge - \tau $ *almost surely* (*a*.*s*.).

### Proof

Since the coefficients of model (3) satisfy the local Lipschitz condition, for any given initial value $(S(\theta ),I(\theta ),R(\theta )) \in \mathbb{R}_{ +}^{3}$, $\theta \in [ - \tau ,0]$, there exists a unique local solution $(S(t), I(t), R(t))$ on $t \in [ - \tau ,\tau _{e})$, where $\tau _{e}$ denotes the explosion time [35]. In order to prove that this solution is global, we need to demonstrate that $\tau _{e} = \infty $ almost surely (briefly a.s.). To that end, let $k_{0} \ge 1$ be sufficiently large so that $S(\theta )$, $I(\theta )$ and $R(\theta )$ ($\theta \in [ - \tau ,0]$) lie within the interval $[\frac{1}{k_{0}}, k_{0}]$. In this case, we define the stopping time as follows for each integer $k \ge k_{0}$:

4 $$ \tau _{k} = \inf \biggl\{ t \in [ - \tau ,\tau _{e} ): \min \bigl\{ S(t),I(t),R(t) \bigr\} \le \frac{1}{k}\mbox{ or }\max \bigl\{ S(t),I(t),R(t) \bigr\} \ge k \biggr\} , $$

where we set $\inf \emptyset = \infty $ (obviously ∅ means the empty set). And notably, $\tau _{k}$ is increasing as $k \to \infty $. Set $\tau _{\infty } = \lim_{k \to \infty } \tau _{k}$, whence $\tau _{\infty } \le \tau _{e}$ a.s. If it is true that $\tau _{\infty } = \infty $ a.s. then $\tau _{e} = \infty $ a.s. and $(S(t),I(t),R(t)) \in \mathbb{R}_{ +}^{3}$ a.s. for all $t \ge - \tau $. That is to say, we only need to demonstrate $\tau _{\infty } = \infty $ a.s. to complete the proof. Instead, suppose that $\tau _{\infty } < \infty $ a.s., then there exists a pair of constants $T > 0$ and $0 < \varepsilon < 1$ such that $\mathrm{P} \{ \tau _{\infty } \le T \} > \varepsilon $. Therefore, there exists an integer $k_{1} \ge k_{0}$ such that $\mathrm{P} \{ \tau _{k} \le T \} \ge \varepsilon $ for all $k \ge k_{1}$.

Define a $C^{2}$-function $V_{1}:\mathbb{R}_{ +}^{2} \to \mathbb{R}_{ +}$ by

5 $$ \begin{aligned} V_{1}\bigl(S(t),I(t),R(t)\bigr) ={}& e^{ - \mu \tau } \biggl(S(t) - m - m\ln \frac{S(t)}{m}\biggr) + \biggl(I(t) - n - n\ln \frac{I(t)}{n}\biggr) \\ &{}+ \bigl(R(t) - 1 - \ln R(t)\bigr) + e^{ - \mu \tau } \int _{t - \tau }^{t} \frac{\beta S(s)I(s)}{1 + \alpha I(s)} \,ds, \end{aligned} $$

where *m* and *n* are two positive constants to be confirmed later. And the nonnegativity of the function (5) can be shown from $u - 1 - \ln u \ge 0$ for $\forall u > 0$.

For any case where ${T} > 0$, $k \ge k_{0}$ and $0 \le t \le \tau _{k} \wedge {T} = \min \{ \tau _{k},{T} \} $, where ∧ denotes the infimum, if $(S(t),I(t),R(t)) \in \mathbb{R}_{ +}^{3}$, by Itô’s formula, we have

6 $$ \begin{aligned} dV_{1}\bigl(S(t),I(t),R(t)\bigr) ={}& LV_{1}\bigl(S(t),I(t),R(t)\bigr)\,dt + e^{ - \mu \tau } \sigma _{1}\bigl(S(t) - m\bigr)\,dB_{1}(t) \\ &{}+ \sigma _{2}\bigl(I(t) - n\bigr)\,dB_{2}(t) + \sigma _{3}\bigl(R(t) - 1\bigr)\,dB_{3}(t), \end{aligned} $$

where $LV_{1}:\mathbb{R}_{ +}^{3} \to \mathbb{R}_{ +}$ is defined by

$$\begin{aligned}& LV_{1}\bigl(S(t),I(t),R(t)\bigr) \\& \quad = e^{ - \mu \tau } \biggl(1 - \frac{m}{S(t)}\biggr)\biggl[(1 - p)b - \frac{\beta S(t)I(t)}{1 + \alpha I(t)} - \mu S(t)\biggr] + \frac{1}{2}me^{ - \mu \tau } \sigma _{1}^{2} \\& \qquad {}+ \biggl(1 - \frac{n}{I(t)}\biggr)\biggl[e^{ - \mu \tau } \frac{\beta S(t - \tau )I(t - \tau )}{1 + \alpha I(t - \tau )} - \eta I(t) \bigl(I(t) + R(t)\bigr) - \mu I(t)\biggr] + \frac{n}{2}\sigma _{2}^{2} \\& \qquad {}+ \biggl(1 - \frac{1}{R(t)}\biggr)\bigl[pb + \eta I(t) \bigl(I(t) + R(t)\bigr) - \mu R(t)\bigr] + \frac{1}{2}\sigma _{3}^{2} \\& \qquad {}+ e^{ - \mu \tau } \frac{\beta S(t)I(t)}{1 + \alpha I(t)} - e^{ - \mu \tau } \frac{\beta S(t - \tau )I(t - \tau )}{1 + \alpha I(t - \tau )} \\& \quad = e^{ - \mu \tau } (1 - p)b - e^{ - \mu \tau } \mu S(t) - e^{ - \mu \tau } \frac{m(1 - p)b}{S(t)} + e^{ - \mu \tau } \frac{m\beta I(t)}{1 + \alpha I(t)} + m\mu e^{ - \mu \tau } \\& \qquad {}+ \frac{m}{2}\sigma _{1}^{2}e^{ - \mu \tau } - \mu I(t) - e^{ - \mu \tau } \frac{n\beta S(t - \tau )I(t - \tau )}{I(t)(1 + \alpha I(t - \tau ))} + n\eta I(t) + n\eta R(t) \\& \qquad {}+ n\mu + \frac{n}{2}\sigma _{2}^{2} + pb - \mu R(t) - \frac{pb}{R(t)} - \frac{\eta I^{2}(t)}{R(t)} - \eta I(t) + \mu + \frac{1}{2}\sigma _{3}^{2} \\& \quad \le e^{ - \mu \tau } (1 - p)b + pb + m\mu e^{ - \mu \tau } + n\mu + \mu + \frac{m}{2}\sigma _{1}^{2}e^{ - \mu \tau } + \frac{n}{2}\sigma _{2}^{2} + \frac{1}{2}\sigma _{3}^{2} \\& \qquad {}+ e^{ - \mu \tau } \frac{m\beta I(t)}{1 + \alpha I(t)} + (n\eta - \eta - \mu )I(t) + (n\eta - \mu )R(t) \\& \quad \le e^{ - \mu \tau } (1 - p)b + pb + m\mu e^{ - \mu \tau } + n\mu + \mu + \frac{m}{2}\sigma _{1}^{2}e^{ - \mu \tau } + \frac{n}{2}\sigma _{2}^{2} + \frac{1}{2}\sigma _{3}^{2} \\& \qquad {}+ \bigl(e^{ - \mu \tau } m\beta + n\eta - \eta - \mu \bigr)I(t) + (n \eta - \mu )R(t). \end{aligned}$$

Choose $m = \frac{\eta }{\beta } e^{\mu \tau }$ and $n = \frac{\mu }{\eta } $ such that $e^{ - \mu \tau } m\beta + n\eta - \eta - \mu = 0$ and $n\eta - \mu = 0$, we get

$$ LV_{1}(S,I,R) \le e^{ - \mu \tau } (1 - p)b + pb + \biggl( \frac{\eta }{\beta } + \frac{\mu }{\eta } + 1\biggr)\mu + \frac{\eta }{2\beta } \sigma _{1}^{2}e^{ - \mu \tau } + \frac{\mu }{2\eta } \sigma _{2}^{2} + \frac{1}{2}\sigma _{3}^{2}: = \tilde{ \mathrm{K}}, $$

where K̃ is a positive constant. Hence, we get

$$\begin{aligned}& \int _{0}^{\tau _{k} \wedge T} dV_{1} \bigl(S(t),I(t),R(t)\bigr) \\& \quad \le \int _{0}^{\tau _{k} \wedge T} \tilde{\mathrm{K}} \,dt + \sigma _{1} \int _{0}^{\tau _{k} \wedge T} \biggl(S - \frac{\eta }{\beta } e^{\mu \tau } \biggr) \,dB_{1}(t) \\& \qquad {}+ \sigma _{2} \int _{0}^{\tau _{k} \wedge T} \biggl(I - \frac{\mu }{\eta } \biggr) \,dB_{2}(t) + \sigma _{3} \int _{0}^{\tau _{k} \wedge T} (R - 1) \,dB_{3}(t). \end{aligned}$$

Then taking the expectation we obtain

$$\begin{aligned} 0 \le \mathrm{E}\bigl[V_{1}\bigl(S(\tau _{k} \wedge T),I(\tau _{k} \wedge T),R(\tau _{k} \wedge T)\bigr) \bigr] &\le V_{1}\bigl(S(0),I(0),R(0)\bigr) + \tilde{\mathrm{K}} \mathrm{E}[\tau _{k} \wedge T] \\ &\le V_{1}\bigl(S(0),I(0),R(0)\bigr) + \tilde{\mathrm{K}}T. \end{aligned}$$

We define $\Omega _{k} = \{ \tau _{k} \le T \} $ for any $k \ge k_{1}$, then by (4) we get $\mathrm{P}(\Omega _{k}) \ge \varepsilon $. Note that some component of $S(\tau _{k},\omega )$, $I(\tau _{k},\omega )$ or $R(\tau _{k},\omega )$ equals either $\frac{1}{k}$ or *k* for each $\omega \in \Omega _{k}$. That is to say, we have

$$ V_{1}\bigl(S(\tau _{k},\omega ),I(\tau _{k},\omega ),R(\tau _{k},\omega )\bigr) \ge (k - 1 - \ln k) \wedge \biggl(\frac{1}{k} - 1 + \ln k\biggr). $$

Combining it with (6), we get

$$\begin{aligned} V_{1}\bigl(S(0),I(0),R(0)\bigr) + \tilde{\mathrm{K}}T &\ge \mathrm{E}\bigl[\mathrm{I}_{\Omega _{k}}(\omega )V\bigl(S(\tau _{k},\omega ),I(\tau _{k},\omega ),R(\tau _{k},\omega )\bigr)\bigr] \\ &\ge \varepsilon \biggl[(k - 1 - \ln k) \wedge \biggl(\frac{1}{k} - 1 + \ln k\biggr)\biggr], \end{aligned}$$

where $\mathrm{I}_{\Omega _{k}}$ means the indicator function of $\Omega _{k}$. As *k* tends to infinity, we find the contradiction in the expression

$$ \infty > V_{1}\bigl(S(0),I(0),R(0)\bigr) + \tilde{\mathrm{K}}T = \infty , $$

therefore, we must have $\tau _{\infty } = + \infty $ a.s., which means that $S(t)$, $I(t)$ and $R(t)$ will not explode in a finite time with probability one. This completes the proof. □

## The dynamic properties around the rumor-free equilibrium

If $R_{0} < 1$, that is, $e^{ - \mu \tau } \beta (1 - p)b \le \eta pb + \mu ^{2}$, the rumor-free equilibrium $P_{0}$ of the model (1) has global asymptotic stability in the invariant set Γ. Nevertheless, $P_{0}$ is not the equilibrium of the model (3). In this section, we study the asymptotic behavior of the global solution $(S(t), I(t), R(t))$around the rumor-free equilibrium $P_{0} = (\frac{(1 - p)b}{\mu },0,\frac{pb}{\mu } )$.

### Theorem 4.1

*Let* $(S(t), I(t), R(t))$ *is an arbitrary solution of model* (3), *for any* $(S(\theta ),I(\theta ), R(\theta )) \in \mathbb{R}_{3}^{ +}$, $\theta \in [ - \tau ,0]$. *If* $R_{0} < 1$ *and the following conditions are satisfied*:

$$ \sigma _{1}^{2} < \frac{\mu ^{2}}{\eta b + \mu ^{2}},\qquad \sigma _{2}^{2} < 2\mu ,\qquad \biggl(\frac{2\mu ^{2}}{\eta b} + 1 \biggr)\sigma _{3}^{2} < \frac{\mu ^{3}}{\eta b}, $$

*then the solution of the model* (3) *has the asymptotic behavior*

7 $$ \lim_{t \to \infty } \sup \frac{1}{t}\mathrm{E} \biggl\{ \int _{0}^{t} \biggl[\biggl(S(u) - \frac{(1 - p)b}{\mu } \biggr)^{2} + I^{2}(u) + \biggl(R(u) - \frac{pb}{\mu } \biggr)^{2}\biggr]\,dt \biggr\} \le \frac{M_{1}}{k_{1}}, $$

*where* $k_{1} = \min \{ 2\mu (\frac{\mu ^{2}}{\eta b + \mu ^{2}} - \sigma _{1}^{2})e^{ - 2\mu \tau },(2\mu - \sigma _{2}^{2}),2(\frac{\mu ^{3}}{\eta b} - \frac{2\mu ^{2} + \eta b}{\eta b}\sigma _{3}^{2}) \} $, *and*

$$ M_{1} = \frac{2\sigma _{1}^{2}(1 - p)^{2}b^{2}e^{ - 2\mu \tau }}{\mu ^{2}} + \frac{2\sigma _{3}^{2}p^{2}b^{2}}{\mu ^{2}} + \frac{4(1 - p)^{2}b^{2}e^{ - 2\mu \tau }}{\mu } + \frac{4pb^{2}}{\mu } + \frac{2\sigma _{3}^{2}p^{2}b}{\eta }. $$

### Proof

Define a $C^{2}$-function $V_{2}:\mathbb{R}_{ +}^{3} \to \mathbb{R}_{ +} $ by

8 $$ V_{2}(S,I,R) = \nu _{1} + x\nu _{2} + y\nu _{3} + \nu _{4}, $$

with

$$\begin{aligned}& \nu _{1} = \biggl[e^{ - \mu \tau } \biggl(S(t) - \frac{(1 - p)b}{\mu } \biggr) + I(t + \tau ) + \biggl(R(t + \tau ) - \frac{pb}{\mu } \biggr)\biggr]^{2}, \\& \nu _{2} = e^{ - \mu \tau } S(t) + I(t + \tau ), \\& \nu _{3} = \biggl(R(t + \tau ) - \frac{pb}{\mu } \biggr)^{2}, \\& \nu _{4} = \bigl(2\mu - \sigma _{2}^{2} \bigr) \int _{t}^{t + \tau } I^{2}(u) \,du + 2 \biggl(\frac{\mu ^{3}}{\eta b} - \frac{2\mu ^{2} + \eta b}{\eta b}\sigma _{3}^{2} \biggr) \int _{t}^{t + \tau } \biggl(R(u) - \frac{pb}{\mu } \biggr)^{2} \,du, \end{aligned}$$

where *x*, *y* are two positive real constants to be chosen as follows.

According to Itô’s formula, we obtain:

For $\nu _{1} = [e^{ - \mu \tau } (S(t) - \frac{(1 - p)b}{\mu } ) + I(t + \tau ) + (R(t + \tau ) - \frac{pb}{\mu } )]^{2}$,

9 $$ \begin{aligned} d\nu _{1} ={}& L\nu _{1}\,dt + 2 \biggl[e^{ - \mu \tau } \biggl(S(t) - \frac{(1 - p)b}{\mu } \biggr) + I(t + \tau ) + \biggl(R(t + \tau ) - \frac{pb}{\mu } \biggr)\biggr] \\ &{}\times \bigl[e^{ - \mu \tau } \sigma _{1}S(t) \,dB_{1}(t) + \sigma _{2}I(t + \tau ) \,dB_{2}(t) + \sigma _{3}R(t + \tau )\,dB_{3}(t) \bigr], \end{aligned} $$

where

10 $$\begin{aligned} L\nu _{1} ={}& 2\biggl[e^{ - \mu \tau } \biggl(S(t) - \frac{(1 - p)b}{\mu } \biggr) + I(t + \tau ) + \biggl(R(t + \tau ) - \frac{pb}{\mu } \biggr)\biggr] \times \biggl\{ e^{ - \mu \tau } \biggl[(1 - p)b \\ &{}- \frac{\beta S(t)I(t)}{1 + \alpha I(t)} - \mu S(t)\biggr] + \biggl[e^{ - \mu \tau } \frac{\beta S(t)I(t)}{1 + \alpha I(t)} - \eta I(t + \tau ) \bigl(I(t + \tau ) + R(t + \tau )\bigr) \\ &{}- \mu I(t + \tau )\biggr] + \bigl[pb + \eta I(t + \tau ) \bigl(I(t + \tau ) + R(t + \tau )\bigr) - \mu R(t + \tau ) \bigr]\biggr\} \\ &{}+ e^{ - 2\mu \tau } \sigma _{1}^{2}S^{2}(t) + \sigma _{2}^{2}I^{2}(t + \tau ) + \sigma _{3}^{2}R^{2}(t + \tau ) \\ ={}& 2\biggl[e^{ - \mu \tau } \biggl(S(t) - \frac{(1 - p)b}{\mu } \biggr) + I(t + \tau ) + \biggl(R(t + \tau ) - \frac{pb}{\mu } \biggr)\biggr] \\ &{}\times \bigl[e^{ - \mu \tau } (1 - p)b + pb - \mu e^{ - \mu \tau } S(t) - \mu I(t + \tau ) - \mu R(t + \tau )\bigr] \\ &{}+ e^{ - 2\mu \tau } \sigma _{1}^{2}S^{2}(t) + \sigma _{2}^{2}I^{2}(t + \tau ) + \sigma _{3}^{2}R^{2}(t + \tau ) \\ ={}& 2\biggl[e^{ - \mu \tau } \biggl(S(t) - \frac{(1 - p)b}{\mu } \biggr) + I(t + \tau ) + \biggl(R(t + \tau ) - \frac{pb}{\mu } \biggr)\biggr] \\ &{}\times \bigl[e^{ - \mu \tau } (1 - p)b + pb - \mu e^{ - \mu \tau } S(t) - \mu I(t + \tau ) - \mu R(t + \tau )\bigr] \\ &{}+ e^{ - 2\mu \tau } \sigma _{1}^{2}S^{2}(t) + \sigma _{2}^{2}I^{2}(t + \tau ) + \sigma _{3}^{2}R^{2}(t + \tau ) \\ ={}& 2\biggl[e^{ - \mu \tau } \biggl(S(t) - \frac{(1 - p)b}{\mu } \biggr) + I(t + \tau ) + \biggl(R(t + \tau ) - \frac{pb}{\mu } \biggr)\biggr] \\ &{}\times \biggl[ - \mu e^{ - \mu \tau } \biggl(S(t) - \frac{(1 - p)b}{\mu } \biggr) - \mu I(t + \tau ) - \mu \biggl(R(t + \tau ) - \frac{pb}{\mu } \biggr)\biggr] \\ &{}+ e^{ - 2\mu \tau } \sigma _{1}^{2}S^{2}(t) + \sigma _{2}^{2}I^{2}(t + \tau ) + \sigma _{3}^{2}R^{2}(t + \tau ) \\ ={}& - 2\mu e^{ - 2\mu \tau } \biggl(S(t) - \frac{(1 - p)b}{\mu } \biggr)^{2} - 2\mu I^{2}(t + \tau ) - 2\mu \biggl(R(t + \tau ) - \frac{pb}{\mu } \biggr)^{2} \\ &{}- 4\mu e^{ - \mu \tau } \biggl(S(t) - \frac{(1 - p)b}{\mu } \biggr)I(t + \tau ) \\ &{}- 4\mu e^{ - \mu \tau } \biggl(S(t) - \frac{(1 - p)b}{\mu } \biggr) \biggl(R(t + \tau ) - \frac{pb}{\mu } \biggr) \\ &{}- 4\mu I(t + \tau ) \biggl(R(t + \tau ) - \frac{pb}{\mu } \biggr) + e^{ - 2\mu \tau } \sigma _{1}^{2}S^{2}(t) + \sigma _{2}^{2}I^{2}(t + \tau ) + \sigma _{3}^{2}R^{2}(t + \tau ). \end{aligned}$$

Using the inequality $(a + b)^{2} \le 2a^{2} + 2b^{2}$ for all $a, b \in R$, we have

11 $$\begin{aligned} L\nu _{1} \le{}& {-} 2\bigl(\mu - \sigma _{1}^{2}\bigr)e^{ - 2\mu \tau } \biggl(S(t) - \frac{(1 - p)b}{\mu } \biggr)^{2} - \bigl(2\mu - \sigma _{2}^{2}\bigr)I^{2}(t + \tau ) \\ &{}- 2\bigl(\mu - \sigma _{3}^{2}\bigr) \biggl(R(t + \tau ) - \frac{pb}{\mu } \biggr)^{2} + 4(1 - p)be^{ - \mu \tau } I(t + \tau ) \\ &{}- 4\mu e^{ - \mu \tau } \biggl(S(t) - \frac{(1 - p)b}{\mu } \biggr) \biggl(R(t + \tau ) - \frac{pb}{\mu } \biggr) \\ &{}- 4\mu I(t + \tau ) \biggl(R(t + \tau ) - \frac{pb}{\mu } \biggr) + e^{ - 2\mu \tau } \frac{2\sigma _{1}^{2}(1 - p)^{2}b^{2}}{\mu ^{2}} + \frac{2\sigma _{3}^{2}p^{2}b^{2}}{\mu ^{2}}. \end{aligned}$$

For $\nu _{2} = e^{ - \mu \tau } S(t) + I(t + \tau )$,

12 $$ d\nu _{2} = L\nu _{2}\,dt + e^{ - \mu \tau } \sigma _{1}S(t)\,dB_{1}(t) + \sigma _{2}I(t + \tau )\,dB_{2}(t), $$

where

13 $$ \begin{aligned} L\nu _{2} ={}& e^{ - \mu \tau } (1 - p)b - e^{ - \mu \tau } \frac{\beta S(t)I(t)}{1 + \alpha I(t)} - \mu e^{ - \mu \tau } S(t) \\ &{}+ e^{ - \mu \tau } \frac{\beta S(t)I(t)}{1 + \alpha I(t)} - \eta I(t + \tau ) \bigl(I(t + \tau ) + R(t + \tau )\bigr) - \mu I(t + \tau ) \\ ={}& e^{ - \mu \tau } (1 - p)b - \mu e^{ - \mu \tau } S(t) - \mu I(t + \tau ) - \eta I(t + \tau ) \bigl(I(t + \tau ) + R(t + \tau )\bigr) \\ \le{}& e^{ - \mu \tau } (1 - p)b - \mu I(t + \tau ). \end{aligned} $$

For $\nu _{3} = (R(t + \tau ) - \frac{pb}{\mu } )^{2}$,

14 $$ d\nu _{3} = L\nu _{3}\,dt + 2\sigma _{3}R(t + \tau ) \biggl(R(t + \tau ) - \frac{pb}{\mu } \biggr)\,dB_{3}(t), $$

where

15 $$ \begin{aligned} L\nu _{3} ={}& 2\biggl(R(t + \tau ) - \frac{pb}{\mu } \biggr)\bigl[pb + \eta I(t + \tau ) \bigl(I(t + \tau ) + R(t + \tau )\bigr) - \mu R(t + \tau )\bigr] \\ &{}+ \sigma _{3}^{2}R^{2}(t + \tau ) \\ ={}& 2\biggl(R(t + \tau ) - \frac{pb}{\mu } \biggr)\biggl[ - \mu \biggl(R(t + \tau ) - \frac{pb}{\mu } \biggr) + \eta I(t + \tau ) \bigl(I(t + \tau ) + R(t + \tau )\bigr)\biggr] \\ &{}+ \sigma _{3}^{2}R^{2}(t + \tau ) \\ ={}& {-} 2\mu \biggl(R(t + \tau ) - \frac{pb}{\mu } \biggr)^{2} + 2\eta I(t + \tau ) \biggl(R(t + \tau ) - \frac{pb}{\mu } \biggr) \bigl(I(t + \tau ) + R(t + \tau )\bigr) \\ &{}+ \sigma _{3}^{2}R^{2}(t + \tau ) \\ \le{}& {-} 2\bigl(\mu - \sigma _{3}^{2}\bigr) \biggl(R(t + \tau ) - \frac{pb}{\mu } \biggr)^{2} + \frac{2\eta b}{\mu } I(t + \tau ) \biggl(R(t + \tau ) - \frac{pb}{\mu } \biggr) \\ &{}+ \frac{2\eta pb^{3}}{\mu ^{3}} + \frac{\sigma _{3}^{2}p^{2}b^{2}}{\mu ^{2}}. \end{aligned} $$

For $\nu _{4} = (2\mu - \sigma _{2}^{2})\int _{t}^{t + \tau } I^{2}(u) \,du + 2(\frac{\mu ^{3}}{\eta b} - \frac{2\mu ^{2} + \eta b}{\eta b}\sigma _{3}^{2})\int _{t}^{t + \tau } (R(u) - \frac{pb}{\mu } )^{2} \,du$,

16 $$ \begin{aligned} d\nu _{4} ={}& \bigl[\bigl(2\mu - \sigma _{2}^{2}\bigr) \bigl(I^{2}(t + \tau ) - I^{2}(t)\bigr)\bigr]\,dt \\ &{}+ \biggl[2\biggl(\frac{\mu ^{3}}{\eta b} - \frac{2\mu ^{2} + \eta b}{\eta b}\sigma _{3}^{2}\biggr) \biggl(\biggl(R(t + \tau ) - \frac{pb}{\mu } \biggr)^{2} - \biggl(R(t) - \frac{pb}{\mu } \biggr)^{2}\biggr)\biggr]\,dt. \end{aligned} $$

Since $V_{2}(S,I,R) = \nu _{1} + x\nu _{2} + y\nu _{3} + \nu _{4}$, substituting Eq. (9)–(16) into $V_{2}$, we have

17 $$ \begin{aligned} dV_{2} ={}& LV_{2}\,dt + e^{ - \mu \tau } \sigma _{1}S(t)\biggl[2e^{ - \mu \tau } \biggl(S(t) - \frac{(1 - p)b}{\mu } \biggr) + 2I(t + \tau ) \\ &{}+ 2\biggl(R(t + \tau ) - \frac{pb}{\mu } \biggr) + x\biggr] \,dB_{1}(t) + \sigma _{2}I(t + \tau ) \biggl[2e^{ - \mu \tau } \biggl(S(t) - \frac{(1 - p)b}{\mu } \biggr) \\ &{}+ 2I(t + \tau ) + 2\biggl(R(t + \tau ) - \frac{pb}{\mu } \biggr) + x \biggr]\,dB_{2}(t) \\ &{}+ \sigma _{3}R(t + \tau ) \biggl[2e^{ - \mu \tau } \biggl(S(t) - \frac{(1 - p)b}{\mu } \biggr) \\ &{}+ 2I(t + \tau ) + 2(y + 1) \biggl(R(t + \tau ) - \frac{pb}{\mu } \biggr)\biggr]\,dB_{3}(t), \end{aligned} $$

where

18 $$ \begin{aligned} LV_{2} \le{}& {-} 2\bigl(\mu - \sigma _{1}^{2}\bigr)e^{ - 2\mu \tau } \biggl(S(t) - \frac{(1 - p)b}{\mu } \biggr)^{2} - \bigl(2\mu - \sigma _{2}^{2}\bigr)I^{2}(t + \tau ) \\ &{}- 2(y + 1) \bigl(\mu - \sigma _{3}^{2}\bigr) \biggl(R(t + \tau ) - \frac{pb}{\mu } \biggr)^{2} + \bigl[4(1 - p)be^{ - \mu \tau } - x\mu \bigr]I(t + \tau ) \\ &{}- 4\mu e^{ - \mu \tau } \biggl(S(t) - \frac{(1 - p)b}{\mu } \biggr) \biggl(R(t + \tau ) - \frac{pb}{\mu } \biggr) \\ &{}+ \biggl(\frac{2\eta by}{\mu } - 4\mu \biggr)I(t + \tau ) \biggl(R(t + \tau ) - \frac{pb}{\mu } \biggr) + e^{ - 2\mu \tau } \frac{2\sigma _{1}^{2}(1 - p)^{2}b^{2}}{\mu ^{2}} \\ &{}+ \frac{2\sigma _{3}^{2}p^{2}b^{2}}{\mu ^{2}} + e^{ - \mu \tau } (1 - p)bx + \biggl( \frac{2\eta pb^{3}}{\mu ^{3}} + \frac{\sigma _{3}^{2}p^{2}b^{2}}{\mu ^{2}}\biggr)y \\ &{}+ \bigl(2\mu - \sigma _{2}^{2}\bigr) \bigl(I^{2}(t + \tau ) - I^{2}(t)\bigr) + [2\bigl[(y + 1) \bigl(\mu - \sigma _{3}^{2}\bigr) - \mu \varepsilon \bigr] \\ &{}\times \biggl(\biggl(R(t + \tau ) - \frac{pb}{\mu } \biggr)^{2} - \biggl(R(t) - \frac{pb}{\mu } \biggr)^{2}\biggr). \end{aligned} $$

By choosing $x = \frac{4(1 - p)be^{ - \mu \tau }}{\mu }$ and $y = \frac{2\mu ^{2}}{\eta b}$, we find that $4(1 - p)be^{ - \mu \tau } - x\mu = 0$ and $\frac{2\eta by}{\mu } - 4\mu = 0$, then we obtain

19 $$ \begin{aligned} LV_{2} \le{}& {-} 2\bigl(\mu - \sigma _{1}^{2}\bigr)e^{ - 2\mu \tau } \biggl(S(t) - \frac{(1 - p)b}{\mu } \biggr)^{2} - \bigl(2\mu - \sigma _{2}^{2}\bigr)I^{2}(t) \\ &{}- 2\biggl(\frac{\mu ^{3}}{\eta b} - \frac{2\mu ^{2} + \eta b}{\eta b}\sigma _{3}^{2}\biggr) \biggl(R(t) - \frac{pb}{\mu } \biggr)^{2} - \frac{2\mu (\mu ^{2} + \eta b)}{\eta b}\biggl(R(t + \tau ) - \frac{pb}{\mu } \biggr)^{2} \\ &{}- 4\mu e^{ - \mu \tau } \biggl(S(t) - \frac{(1 - p)b}{\mu } \biggr) \biggl(R(t + \tau ) - \frac{pb}{\mu } \biggr) + \frac{2\sigma _{1}^{2}(1 - p)^{2}b^{2}e^{ - 2\mu \tau }}{\mu ^{2}} \\ &{}+ \frac{2\sigma _{3}^{2}p^{2}b^{2}}{\mu ^{2}} + \frac{4(1 - p)^{2}b^{2}e^{ - 2\mu \tau }}{\mu } + \frac{4pb^{2}}{\mu } + \frac{2\sigma _{3}^{2}p^{2}b}{\eta } \\ \le{}& {-} 2\mu \biggl(\frac{\mu ^{2}}{\eta b + \mu ^{2}} - \sigma _{1}^{2} \biggr)e^{ - 2\mu \tau } \biggl(S(t) - \frac{(1 - p)b}{\mu } \biggr)^{2} - \bigl(2\mu - \sigma _{2}^{2} \bigr)I^{2}(t) \\ &{}- 2\biggl(\frac{\mu ^{3}}{\eta b} - \frac{2\mu ^{2} + \eta b}{\eta b}\sigma _{3}^{2}\biggr) \biggl(R(t) - \frac{pb}{\mu } \biggr)^{2} + M_{1}, \end{aligned} $$

where in the above inequality we used the Young inequality [36]

$$\begin{aligned} - 4\mu e^{ - \mu \tau } \biggl(S(t) - \frac{(1 - p)b}{\mu } \biggr) \biggl(R(t + \tau ) - \frac{pb}{\mu } \biggr) \le& \frac{2\mu e^{ - 2\mu \tau }}{\varepsilon } \biggl(S(t) - \frac{(1 - p)b}{\mu } \biggr)^{2} \\ &{}+ 2\mu \varepsilon \biggl(R(t + \tau ) - \frac{pb}{\mu } \biggr)^{2}, \end{aligned}$$

where $\varepsilon = \frac{\eta b + \mu ^{2}}{\eta b}$.

Integrating both sides of (19) between 0 and *t* and meanwhile taking expectation, we obtain

$$\begin{aligned} 0 &\le \mathrm{E}V_{2}(t) - \mathrm{E}V_{2}(0) \\ &\le - 2\mu \biggl(\frac{\mu ^{2}}{\eta b + \mu ^{2}} - \sigma _{1}^{2} \biggr)e^{ - 2\mu \tau } \mathrm{E} \int _{0}^{t} \biggl( S(t) - \frac{(1 - p)b}{\mu } \biggr)^{2}\,du - \bigl(2\mu - \sigma _{2}^{2}\bigr)\mathrm{E} \int _{0}^{t} I^{2}(t)\,du \\ &\quad {}- 2\biggl(\frac{\mu ^{3}}{\eta b} - \frac{2\mu ^{2} + \eta b}{\eta b}\sigma _{3}^{2}\biggr)\mathrm{E} \int _{0}^{t} \biggl( R(t) - \frac{pb}{\mu } \biggr)^{2}\,du + M_{1}t. \end{aligned}$$

Let $k_{1} = \min \{ 2\mu (\frac{\mu ^{2}}{\eta b + \mu ^{2}} - \sigma _{1}^{2})e^{ - 2\mu \tau },(2\mu - \sigma _{2}^{2}),2(\frac{\mu ^{3}}{\eta b} - \frac{2\mu ^{2} + \eta b}{\eta b}\sigma _{3}^{2}) \} $, then

$$ \mathrm{E} \biggl\{ \int _{0}^{t} \biggl[\biggl(S(u) - \frac{(1 - p)b}{\mu } \biggr)^{2} + I^{2}(u) + \biggl(R(u) - \frac{pb}{\mu } \biggr)^{2}\biggr]\,dt \biggr\} \le \frac{M_{1}}{k_{1}}t. $$

Consequently,

$$ \lim_{t \to \infty } \sup \frac{1}{t}\mathrm{E} \biggl\{ \int _{0}^{t} \biggl[\biggl(S(u) - \frac{(1 - p)b}{\mu } \biggr)^{2} + I^{2}(u) + \biggl(R(u) - \frac{pb}{\mu } \biggr)^{2}\biggr]\,dt \biggr\} \le \frac{M_{1}}{k_{1}}. $$

 □

### Remark 4.2

If $R_{0} \le 1$ and under the conditions of Theorem 4.1, we conclude that the solution fluctuates around the rumor-free equilibrium.

## The dynamic properties around the rumor-local equilibrium

If $R_{0} > 1$, there exists a rumor-local equilibrium $P^{*} = (S^{*},I^{*},R^{*})$ of model (1), however, it does not denote the equilibrium of model (3). Therefore, we study the asymptotic behavior of the global solution $(S(t), I(t), R(t))$ of model (3) around the rumor-local equilibrium $P^{*}$ in this part.

### Theorem 5.1

*Let* $(S(t), I(t), R(t))$ *be an arbitrary solution of model* (3), *for any* $(S(\theta ),I(\theta ), R(\theta )) \in \mathbb{R}_{3}^{ +}$, $\theta \in [ - \tau ,0]$. *If* $R_{0} > 1$, *consider the following conditions*:

$$\begin{aligned}& \frac{y(\mu ^{2} - \eta b)\mu }{y(\mu ^{2} - \eta b) + 2\mu ^{2}} > \sigma _{1}^{2},\qquad \mu + x \eta > \sigma _{2}^{2},\qquad \biggl(\frac{y}{2} + 2\biggr)\mu > (y + 1)\sigma _{3}^{2}, \\& \mu ^{2} > \eta b,\qquad x = \frac{2\mu (1 + \alpha I^{*})}{\beta },\qquad y = \frac{2\mu (\beta + 1 + \alpha I^{*})}{\eta \beta (I^{*} + R^{*})}. \end{aligned}$$

*Then the solution satisfies*

20 $$ \lim_{t \to \infty } \sup \frac{1}{t}\mathrm{E} \biggl\{ \int _{0}^{t} \bigl[\bigl(S(u) - S^{*}\bigr)^{2} + \bigl(I(u) - I^{*} \bigr)^{2} + \bigl(R(u) - R^{*}\bigr)^{2} \bigr]\,dt \biggr\} \le \frac{M_{2}}{k_{2}}, $$

*where* $k_{2} = \min \{ (\frac{y(\mu ^{2} - \eta b)\mu }{y(\mu ^{2} - \eta b) + 2\mu ^{2}} - \sigma _{1}^{2}),(\mu + x\eta - \sigma _{2}^{2}),((\frac{y}{2} + 2)\mu - (y + 1)\sigma _{3}^{2}) \}$,

$$\begin{aligned} M_{2} =& e^{ - 2\mu \tau } \sigma _{1}^{2}S^{*^{2}} + \sigma _{2}^{2}I^{*^{2}} + \frac{\mu I^{*}(1 + \alpha I^{*})}{\beta } + \frac{2\mu \sigma _{3}^{2}(\beta + 1 + \alpha I^{*})R^{*}}{\eta \beta (I^{*} + R^{*})} \\ &{}+ \frac{(1 - p)be^{ - \mu \tau }}{\mu } \biggl[2\mu \bigl(1 + \alpha I^{*}\bigr) \biggl(\frac{b}{\mu } + I^{*}\biggr) + \eta \biggl( \frac{b^{2}}{\mu ^{2}} + I^{*}R^{*}\biggr)\biggr]. \end{aligned}$$

### Proof

Since $P^{*} = (S^{*},I^{*},R^{*})$ is the rumor-local equilibrium of model (1), we get

$$ \textstyle\begin{cases} (1 - p)b - \frac{\beta S^{*}I^{*}}{1 + \alpha I^{*}} - \mu S^{*} = 0, \\ e^{ - \mu \tau } \frac{\beta S^{*}I^{*}}{1 + \alpha I^{*}} - \lambda I^{*}(I^{*} + R^{*}) - \mu I^{*} = 0, \\ pb + \lambda I^{*}(I^{*} + R^{*}) - \mu R^{*} = 0. \end{cases} $$

We consider the following function:

21 $$ V_{3}(S,I,R) = \upsilon _{1} + x\upsilon _{2} + y\upsilon _{3} + z\upsilon _{4} + \upsilon _{5}, $$

with

$$\begin{aligned}& \upsilon _{1} = \frac{1}{2}\bigl[e^{ - \mu \tau } \bigl(S(t) - S^{*}\bigr) + \bigl(I(t + \tau ) - I^{*} \bigr) + \bigl(R(t + \tau ) - R^{*}\bigr)\bigr]^{2}, \\& \upsilon _{2} = I(t + \tau ) - I^{*} - I^{*} \ln \frac{I(t + \tau )}{I^{*}}, \\& \upsilon _{3} = \frac{1}{2}\bigl(R(t + \tau ) - R^{*}\bigr)^{2}, \\& \upsilon _{4} = e^{ - \mu \tau } S(t) + I(t + \tau ), \\& \upsilon _{5} = \bigl(x\eta + \mu - \sigma _{2}^{2} \bigr) \int _{t}^{t + \tau } \bigl(I(u) - I^{*} \bigr)^{2}\,du \\& \hphantom{\upsilon _{5} ={}}{}+ \biggl[\biggl(\frac{y}{2} + 2\biggr)\mu - (y + 1)\sigma _{3}^{2}\biggr] \int _{t}^{t + \tau } \bigl(R(u) - R^{*} \bigr)^{2} \,du, \end{aligned}$$

where *x*, *y* and *z* are three positive constants to be chosen as follows and $z = \frac{1}{\mu } [\beta x(\frac{b}{\mu } + I^{*}) + \eta y(\frac{b^{2}}{\mu ^{2}} + I^{*}R^{*})]$ which is determined by *x*, *y*. According to Ito’s formula, we get for $\upsilon _{1} = \frac{1}{2}[e^{ - \mu \tau } (S(t) - S^{*}) + (I(t + \tau ) - I^{*}) + (R(t + \tau ) - R^{*})]^{2}$,

22 $$ \begin{aligned} d\upsilon _{1} ={}& L\upsilon _{1}\,dt + \bigl[e^{ - \mu \tau } \bigl(S(t) - S^{*}\bigr) + \bigl(I(t + \tau ) - I^{*}\bigr) + \bigl(R(t + \tau ) - R^{*}\bigr)\bigr] \\ &{}\times \bigl[e^{ - \mu \tau } \sigma _{1}S(t) \,dB_{1}(t) + \sigma _{2}I(t + \tau ) \,dB_{2}(t) + \sigma _{3}R(t + \tau )\,dB_{3}(t) \bigr], \end{aligned} $$

where

23 $$\begin{aligned} L\upsilon _{1} ={}& \bigl[e^{ - \mu \tau } \bigl(S(t) - S^{*}\bigr) + \bigl(I(t + \tau ) - I^{*} \bigr) + \bigl(R(t + \tau ) - R^{*}\bigr)\bigr] \\ &{}\times \bigl[e^{ - \mu \tau } (1 - p)b + pb - \mu e^{ - \mu \tau } S(t) - \mu I(t + \tau ) - \mu R(t + \tau )\bigr] \\ &{}+ \frac{1}{2}e^{ - 2\mu \tau } \sigma _{1}^{2}S^{2}(t) + \frac{1}{2}\sigma _{2}^{2}I^{2}(t + \tau ) + \frac{1}{2}\sigma _{3}^{2}R^{2}(t + \tau ) \\ ={}& {-} \bigl[e^{ - \mu \tau } \bigl(S(t) - S^{*}\bigr) + \bigl(I(t + \tau ) - I^{*}\bigr) + \bigl(R(t + \tau ) - R^{*}\bigr)\bigr] \\ &{}\times \bigl[\mu e^{ - \mu \tau } \bigl(S(t) - S^{*}\bigr) + \mu \bigl(I(t + \tau ) - I^{*}\bigr) + \mu \bigl(R(t + \tau ) - R^{*}\bigr)\bigr] \\ &{}+ \frac{1}{2}e^{ - 2\mu \tau } \sigma _{1}^{2}S^{2}(t) + \frac{1}{2}\sigma _{2}^{2}I^{2}(t + \tau ) + \frac{1}{2}\sigma _{3}^{2}R^{2}(t + \tau ) \\ ={}& {-} \mu e^{ - 2\mu \tau } \bigl(S(t) - S^{*} \bigr)^{2} - \mu \bigl(I(t + \tau ) - I^{*} \bigr)^{2} - \mu \bigl(R(t + \tau ) - R^{*} \bigr)^{2} \\ &{}- 2\mu e^{ - \mu \tau } \bigl(S(t) - S^{*}\bigr) \bigl(I(t + \tau ) - I^{*}\bigr) - 2\mu e^{ - \mu \tau } \bigl(S(t) - S^{*}\bigr) \bigl(R(t + \tau ) - R^{*}\bigr) \\ &{}- 2\mu \bigl(I(t + \tau ) - I^{*}\bigr) \bigl(R(t + \tau ) - R^{*}\bigr) + \frac{1}{2}e^{ - 2\mu \tau } \sigma _{1}^{2}S^{2}(t) \\ &{}+ \frac{1}{2}\sigma _{2}^{2}I^{2}(t + \tau ) + \frac{1}{2}\sigma _{3}^{2}R^{2}(t + \tau ). \end{aligned}$$

Using the inequality $(a + b)^{2} \le 2a^{2} + 2b^{2}$ for all $a,b \in R$, we get

24 $$ \begin{aligned} L\upsilon _{1} \le{}& {-} \bigl(\mu - \sigma _{1}^{2}\bigr)e^{ - 2\mu \tau } \bigl(S(t) - S^{*}\bigr)^{2} - \bigl(\mu - \sigma _{2}^{2} \bigr) \bigl(I(t + \tau ) - I^{*}\bigr)^{2} \\ &{}- \bigl(\mu - \sigma _{3}^{2}\bigr) \bigl(R(t + \tau ) - R^{*}\bigr)^{2} - 2\mu e^{ - \mu \tau } \bigl(S(t) - S^{*}\bigr) \bigl(I(t + \tau ) - I^{*}\bigr) \\ &{}- 2\mu e^{ - \mu \tau } \bigl(S(t) - S^{*}\bigr) \bigl(R(t + \tau ) - R^{*}\bigr) - 2\mu \bigl(I(t + \tau ) - I^{*} \bigr) \bigl(R(t + \tau ) - R^{*}\bigr) \\ &{}+ e^{ - 2\mu \tau } \sigma _{1}^{2}S^{*^{2}} + \sigma _{2}^{2}I^{*^{2}} + \sigma _{3}^{2}R^{*^{2}}. \end{aligned} $$

For $\upsilon _{2} = I(t + \tau ) - I^{*} - I^{*}\ln \frac{I(t + \tau )}{I^{*}}$,

25 $$ d\upsilon _{2} = L\upsilon _{2}\,dt + \sigma _{2}\bigl(I(t + \tau ) - I^{*}\bigr)\,dB_{2}(t), $$

where

26 $$\begin{aligned} L\upsilon _{2} ={}& \biggl(1 - \frac{I^{*}}{I(t + \tau )} \biggr)\biggl[e^{ - \mu \tau } \frac{\beta S(t)I(t)}{1 + \alpha I(t)} - \eta I(t + \tau ) \bigl(I(t + \tau ) + R(t + \tau )\bigr) - \mu I(t + \tau )\biggr] \\ &{}+ \frac{1}{2}\sigma _{2}^{2}I^{*} \\ ={}& \bigl(I(t + \tau ) - I^{*}\bigr)\biggl[e^{ - \mu \tau } \frac{\beta S(t)I(t)}{I(t + \tau )(1 + \alpha I(t))} - \eta \bigl(I(t + \tau ) + R(t + \tau )\bigr) - \mu \biggr] \\ &{}+ \frac{1}{2}\sigma _{2}^{2}I^{*} \\ ={}& \bigl(I(t + \tau ) - I^{*}\bigr)\biggl[e^{ - \mu \tau } \frac{\beta S(t)I(t)}{I(t + \tau )(1 + \alpha I(t))} - e^{ - \mu \tau } \frac{\beta S^{*}}{1 + \alpha I^{*}} \\ &{}- \eta \bigl(I(t + \tau ) + R(t + \tau )\bigr) + \eta \bigl(I^{*} + R^{*}\bigr)\biggr] + \frac{1}{2}\sigma _{2}^{2}I^{*} \\ ={}& \bigl(I(t + \tau ) - I^{*}\bigr)\biggl[e^{ - \mu \tau } \frac{\beta S(t)I(t)}{I(t + \tau )(1 + \alpha I(t))} - e^{ - \mu \tau } \frac{\beta S(t)}{1 + \alpha I^{*}} \\ &{}+ e^{ - \mu \tau } \frac{\beta }{1 + \alpha I^{*}}\bigl(S(t) - S^{*}\bigr) - \eta \bigl(I(t + \tau ) - I^{*}\bigr) - \eta \bigl(R(t + \tau ) - R^{*}\bigr)\biggr] + \frac{1}{2}\sigma _{2}^{2}I^{*} \\ ={}& e^{ - \mu \tau } \beta S(t) \biggl(\frac{I(t)}{I(t + \tau )(1 + \alpha I(t))} - \frac{1}{1 + \alpha I^{*}}\biggr) \bigl(I(t + \tau ) - I^{*}\bigr) \\ &{}+ e^{ - \mu \tau } \frac{\beta }{1 + \alpha I^{*}}\bigl(S(t) - S^{*}\bigr) \bigl(I(t + \tau ) - I^{*}\bigr) - \eta \bigl(I(t + \tau ) - I^{*}\bigr)^{2} \\ &{}- \eta \bigl(I(t + \tau ) - I^{*}\bigr) \bigl(R(t + \tau ) - R^{*}\bigr) + \frac{1}{2}\sigma _{2}^{2}I^{*} \\ \le{}& e^{ - \mu \tau } \beta S(t) \biggl(\frac{I(t)}{1 + \alpha I(t)} + \frac{I^{*}}{1 + \alpha I^{*}}\biggr) + e^{ - \mu \tau } \frac{\beta }{1 + \alpha I^{*}}\bigl(S(t) - S^{*}\bigr) \bigl(I(t + \tau ) - I^{*}\bigr) \\ &{}- \eta \bigl(I(t + \tau ) - I^{*}\bigr)^{2} - \eta \bigl(I(t + \tau ) - I^{*}\bigr) \bigl(R(t + \tau ) - R^{*}\bigr) + \frac{1}{2}\sigma _{2}^{2}I^{*} \\ \le{}& e^{ - \mu \tau } \beta S(t) \bigl(I(t) + I^{*}\bigr) + e^{ - \mu \tau } \frac{\beta }{1 + \alpha I^{*}}\bigl(S(t) - S^{*}\bigr) \bigl(I(t + \tau ) - I^{*}\bigr) \\ &{}- \eta \bigl(I(t + \tau ) - I^{*}\bigr)^{2} - \eta \bigl(I(t + \tau ) - I^{*}\bigr) \bigl(R(t + \tau ) - R^{*}\bigr) + \frac{1}{2}\sigma _{2}^{2}I^{*} \\ \le{}& e^{ - \mu \tau } \beta S(t) \biggl(\frac{b}{\mu } + I^{*}\biggr) + e^{ - \mu \tau } \frac{\beta }{1 + \alpha I^{*}}\bigl(S(t) - S^{*}\bigr) \bigl(I(t + \tau ) - I^{*}\bigr) \\ &{}- \eta \bigl(I(t + \tau ) - I^{*}\bigr)^{2} - \eta \bigl(I(t + \tau ) - I^{*}\bigr) \bigl(R(t + \tau ) - R^{*}\bigr) + \frac{1}{2}\sigma _{2}^{2}I^{*}. \end{aligned}$$

For $\upsilon _{3} = \frac{1}{2}(R(t + \tau ) - R^{*})^{2}$,

27 $$ d\upsilon _{3} = L\upsilon _{3}\,dt + \sigma _{3}\bigl(R(t + \tau ) - R^{*}\bigr)\,dB_{3}(t), $$

where

28 $$ \begin{aligned} L\upsilon _{3} ={}& \bigl(R(t + \tau ) - R^{*}\bigr)\bigl[pb + \eta I(t + \tau ) \bigl(I(t + \tau ) + R(t + \tau )\bigr) - \mu R(t + \tau )\bigr] \\ &{}+ \frac{1}{2}\sigma _{3}^{2}R^{2}(t + \tau ) \\ ={}& \bigl(R(t + \tau ) - R^{*}\bigr)\bigl[ - \mu \bigl(R(t + \tau ) - R^{*}\bigr) + \eta \bigl(I^{*} + R^{*} \bigr) \bigl(I(t + \tau ) - I^{*}\bigr) \\ &{}+ \eta I(t + \tau ) \bigl(I(t + \tau ) - I^{*}\bigr) + \eta I(t + \tau ) \bigl(R(t + \tau ) - R^{*}\bigr)\bigr] + \frac{1}{2} \sigma _{3}^{2}R^{2}(t + \tau ) \\ ={}& {-} \mu \bigl(R(t + \tau ) - R^{*}\bigr)^{2} + \eta \bigl(I^{*} + R^{*}\bigr) \bigl(I(t + \tau ) - I^{*}\bigr) \bigl(R(t + \tau ) - R^{*}\bigr) + \eta I(t + \tau ) \\ &{}\times \bigl(I(t + \tau ) - I^{*}\bigr) \bigl(R(t + \tau ) - R^{*}\bigr) + \eta I(t + \tau ) \bigl(R(t + \tau ) - R^{*}\bigr)^{2} + \frac{1}{2}\sigma _{3}^{2}R^{2}(t + \tau ) \\ \le{}& {-} \biggl(\mu - \frac{\eta b}{\mu } - \sigma _{3}^{2} \biggr) \bigl(R(t + \tau ) - R^{*}\bigr)^{2} + \eta \biggl(\frac{b^{2}}{\mu ^{2}} + I^{*}R^{*}\biggr)I(t + \tau ) \\ &{}+ \eta \bigl(I^{*} + R^{*}\bigr) \bigl(I(t + \tau ) - I^{*}\bigr) \bigl(R(t + \tau ) - R^{*}\bigr) + \sigma _{3}^{2}R^{*^{2}}. \end{aligned} $$

For $\upsilon _{4} = e^{ - \mu \tau } S(t) + I(t + \tau )$,

29 $$ d\upsilon _{4} = L\upsilon _{4}\,dt + e^{ - \mu \tau } \sigma _{1}S(t)\,dB_{1}(t) + \sigma _{2}I(t + \tau )\,dB_{2}(t), $$

where

30 $$ \begin{aligned} L\upsilon _{4} ={}& e^{ - \mu \tau } \biggl[(1 - p)b - \frac{\beta S(t)I(t)}{1 + \alpha I(t)} - \mu S(t)\biggr] + e^{ - \mu \tau } \frac{\beta S(t)I(t)}{1 + \alpha I(t)} \\ &{}- \eta I(t + \tau ) \bigl(I(t + \tau ) + R(t + \tau )\bigr) - \mu I(t + \tau ) \\ ={}& e^{ - \mu \tau } (1 - p)b - \mu e^{ - \mu \tau } S(t) - \mu I(t + \tau ) - \eta I(t + \tau ) \bigl(I(t + \tau ) + R(t + \tau )\bigr) \\ \le{}& e^{ - \mu \tau } (1 - p)b - \mu e^{ - \mu \tau } S(t) - \mu I(t + \tau). \end{aligned} $$

For $\upsilon _{5} = (x\eta + \mu - \sigma _{2}^{2})\int _{t}^{t + \tau } (I(u) - I^{*})^{2}\,du + [(\frac{y}{2} + 2)\mu - (y + 1)\sigma _{3}^{2}]\int _{t}^{t + \tau } (R(u) - R^{*})^{2} \,du$,

31 $$ \begin{aligned} d\upsilon _{5} ={}& \bigl(x\eta + \mu - \sigma _{2}^{2}\bigr)\bigl[\bigl(I(t + \tau ) - I^{*}\bigr)^{2} - \bigl(I(t) - I^{*} \bigr)^{2}\bigr]\,dt \\ &{}+ \biggl[\biggl(\frac{y}{2} + 2\biggr)\mu - (y + 1)\sigma _{3}^{2}\biggr] \bigl[\bigl(R(t + \tau ) - R^{*}\bigr)^{2} - \bigl(R(t) - R^{*} \bigr)^{2}\bigr]\,dt. \end{aligned} $$

Since $V_{3}(S,I,R) = \upsilon _{1} + x\upsilon _{2} + y\upsilon _{3} + z\upsilon _{4} + \upsilon _{5}$, substituting Eqs. (22)–(31) into $V_{3}$, we have

32 $$ \begin{aligned} &dV_{3}(S,I,R) \\ &\quad \le \biggl\{ - \bigl(\mu - \sigma _{1}^{2}\bigr)e^{ - 2\mu \tau } \bigl(S(t) - S^{*}\bigr)^{2} - \bigl(\mu + x\eta - \sigma _{2}^{2}\bigr) \bigl(I(t) - I^{*} \bigr)^{2} \\ &\qquad {}- \frac{y(\mu ^{2} - \eta b) + 2\mu ^{2}}{2\mu } \bigl(R(t + \tau ) - R^{*} \bigr)^{2} - \biggl[\biggl(\frac{y}{2} + 2\biggr)\mu - (y + 1)\sigma _{3}^{2}\biggr] \\ &\qquad {}\times \bigl(R(t) - R^{*}\bigr)^{2} + \biggl( \frac{x\beta }{1 + \alpha I^{*}} - 2\mu \biggr)e^{ - \mu \tau } \bigl(S(t) - S^{*}\bigr) \bigl(I(t + \tau ) - I^{*}\bigr) \\ &\qquad {}- 2\mu e^{ - \mu \tau } \bigl(S(t) - S^{*}\bigr) \bigl(R(t + \tau ) - R^{*}\bigr) + \bigl[y\eta \bigl(I^{*} + R^{*}\bigr) - 2\mu \bigr] \\ &\qquad {}\times \bigl(I(t + \tau ) - I^{*}\bigr) \bigl(R(t + \tau ) - R^{*}\bigr) - e^{ - \mu \tau } \eta y\biggl(\frac{b^{2}}{\mu ^{2}} + I^{*}R^{*}\biggr)S(t) \\ &\qquad {}- \beta x\biggl(\frac{b}{\mu } + I^{*}\biggr)I(t + \tau ) + e^{ - 2\mu \tau } \sigma _{1}^{2}S^{*^{2}} + \sigma _{2}^{2}I^{*^{2}} + \frac{x}{2} \sigma _{2}^{2}I^{*} + \frac{x\eta b}{\mu } \bigl(I^{*} + R^{*}\bigr) \\ &\qquad {}+ \sigma _{3}^{2}R^{*^{2}} + y\sigma _{3}^{2}R^{*^{2}} + \frac{1}{\mu } \biggl[\beta x\biggl(\frac{b}{\mu } + I^{*}\biggr) + \eta y \biggl(\frac{b^{2}}{\mu ^{2}} + I^{*}R^{*}\biggr) \biggr]e^{ - \mu \tau } (1 - p)b \biggr\} \,dt \\ &\qquad {}+ e^{ - \mu \tau } \sigma _{1}S(t)\bigl[e^{ - \mu \tau } \bigl(S(t) - S^{*}\bigr) + \bigl(I(t + \tau ) - I^{*} \bigr) + \bigl(R(t + \tau ) - R^{*}\bigr)+ z\bigr] \,dB_{1}(t) \\ &\qquad {}+ \bigl\{ \sigma _{2}I(t + \tau )\bigl[e^{ - \mu \tau } \bigl(S(t) - S^{*}\bigr) + \bigl(I(t + \tau ) - I^{*} \bigr) \\ &\qquad {}+ \bigl(R(t + \tau ) - R^{*}\bigr) + z\bigr] + x\sigma _{2}\bigl(I(t + \tau ) - I^{*}\bigr)\bigr\} \,dB_{2}(t) + \bigl\{ \sigma _{3}R(t + \tau ) \\ &\qquad {}\times \bigl[e^{ - \mu \tau } \bigl(S(t) - S^{*}\bigr) + \bigl(I(t + \tau ) - I^{*}\bigr) + (1 + y) \bigl(R(t + \tau ) - R^{*}\bigr)\bigr]\bigr\} \,dB_{3}(t). \end{aligned} $$

Choosing $x = \frac{2\mu (1 + \alpha I^{*})}{\beta } $ and $y = \frac{2\mu }{\eta (I^{*} + R^{*})}$ such that $\frac{x\beta }{1 + \alpha I^{*}} - 2\mu = 0$ and $y\eta (I^{*} + R^{*}) - x\eta - 2\mu = 0$. Hence, we get

33 $$ \begin{aligned} LV_{3}(S,I,R) \le{}& {-} \bigl(\mu - \sigma _{1}^{2}\bigr)e^{ - 2\mu \tau } \bigl(S(t) - S^{*}\bigr)^{2} - \bigl(\mu + x\eta - \sigma _{2}^{2}\bigr) \bigl(I(t) - I^{*} \bigr)^{2} \\ &{}- \frac{y(\mu ^{2} - \eta b)\mu }{y(\mu ^{2} - \eta b) + 2\mu ^{2}}\bigl(R(t + \tau ) - R^{*} \bigr)^{2} - \biggl[\biggl(\frac{y}{2} + 2\biggr)\mu - (y + 1)\sigma _{3}^{2}\biggr] \\ &{}\times \bigl(R(t) - R^{*}\bigr)^{2} - 2\mu e^{ - \mu \tau } \bigl(S(t) - S^{*}\bigr) \bigl(R(t + \tau ) - R^{*}\bigr) + M_{2} \\ \le{}& {-} \biggl(\frac{y(\mu ^{2} - \eta b)\mu }{y(\mu ^{2} - \eta b) + 2\mu ^{2}} - \sigma _{1}^{2} \biggr)e^{ - 2\mu \tau } \bigl(S(t) - S^{*}\bigr)^{2} - \bigl(\mu + x\eta - \sigma _{2}^{2}\bigr) \\ &{}\times \bigl(I(t) - I^{*}\bigr)^{2} - \biggl[\biggl( \frac{y}{2} + 2\biggr)\mu - (y + 1)\sigma _{3}^{2} \biggr]\bigl(R(t) - R^{*}\bigr)^{2} + M_{2}, \end{aligned} $$

where in the above inequality we used the Young inequality [36]

$$ - 2\mu e^{ - \mu \tau } \bigl(S(t) - S^{*}\bigr) \bigl(R(t + \tau ) - R^{*}\bigr) \le \frac{\mu }{\varepsilon } e^{ - 2\mu \tau } \bigl(S(t) - S^{*}\bigr)^{2} + \varepsilon \mu \bigl(R(t + \tau ) - R^{*}\bigr)^{2}, $$

where $\varepsilon = \frac{y(\mu - \eta b) + 2\mu ^{2}}{2\mu ^{2}}$.

Integrating both sides of (33) between 0 and *t* and meanwhile taking expectation, we obtain

$$\begin{aligned} 0 &\le \mathrm{E}V_{3}(t) - \mathrm{E}V_{3}(0) \\ &\le - \biggl(\frac{y(\mu ^{2} - \eta b)\mu }{y(\mu ^{2} - \eta b) + 2\mu ^{2}} - \sigma _{1}^{2} \biggr)\mathrm{E} \int _{0}^{t} \bigl( S(t) - S^{*} \bigr)^{2}\,du \\ &\quad {}- \bigl(\mu + x\eta - \sigma _{2}^{2}\bigr) \mathrm{E} \int _{0}^{t} \bigl(I(t) - I^{*} \bigr)^{2} \,du \\ &\quad {}- \biggl[\biggl(\frac{y}{2} + 2\biggr)\mu - (y + 1)\sigma _{3}^{2}\biggr]\mathrm{E} \int _{0}^{t} \bigl( R(t) - R^{*} \bigr)^{2}\,du + M_{2}t. \end{aligned}$$

Let $k_{2} = \min \{ (\frac{y(\mu ^{2} - \eta b)\mu }{y(\mu ^{2} - \eta b) + 2\mu ^{2}} - \sigma _{1}^{2}),(\mu + x\eta - \sigma _{2}^{2}),((\frac{y}{2} + 2)\mu - (y + 1)\sigma _{3}^{2}) \}$.

Then

$$ \mathrm{E} \biggl\{ \int _{0}^{t} \bigl[\bigl(S(u) - S^{*}\bigr)^{2} + \bigl(I(u) - I^{*} \bigr)^{2} + \bigl(R(u) - R^{*}\bigr)^{2} \bigr]\,dt \biggr\} \le \frac{M_{2}}{k_{2}}t. $$

Consequently,

$$ \lim_{t \to \infty } \sup \frac{1}{t}\mathrm{E} \biggl\{ \int _{0}^{t} \bigl[\bigl(S(u) - S^{*}\bigr)^{2} + \bigl(I(u) - I^{*} \bigr)^{2} + \bigl(R(u) - R^{*}\bigr)^{2} \bigr]\,dt \biggr\} \le \frac{M_{2}}{k_{2}}. $$

 □

### Remark 5.2

If $R_{0} > 1$, according to Theorem 5.1, we can conclude that the solution of model (3) will fluctuate around the rumor-local equilibrium.

## Numerical simulations

There is no point in just modeling and theorizing to deal with real problems in the absence of effective numerical algorithms and computing equipment [37, 38]. In addition, the model does not have exact solutions in closed form, so it is more appropriate to analyze solutions by numerical simulations. In this section, we shall use Matlab to carry out some numerical simulations to illustrate the availability of the above analytical results. In terms of the above Theorems 4.1 and 5.1, the following part covers some simulation results. We set the time step $\Delta t = 10^{-3}$.

Firstly, we consider the initial value $(S(0),I(0),R(0)) = (0.7,0.3,0.1)$ with related parameter values: $p = 0.32$, $b = 0.5$, $\mu = 0.25$, $\beta = 0.45$, $\alpha = 0.1$, $\eta = 0.1$, $\tau = \ln 32$, $\sigma _{i} = 0.1$ ($i = 1,2,3$). In this case, $R_{0} = 0.819 < 1$ and the conditions in Theorem 4.1 are satisfied:

$$\begin{aligned}& \sigma _{1}^{2} = 0.01 < 0.56 = \frac{\mu ^{2}}{\eta b + \mu ^{2}}, \qquad \sigma _{2}^{2} = 0.01 < 0.52 = \mu , \\& \frac{2\mu ^{2} + \eta b}{\eta b}\sigma _{3}^{2} = 0.0225 < 0.3125 = \frac{\mu ^{3}}{\eta b}. \end{aligned}$$

**Figure 1:**
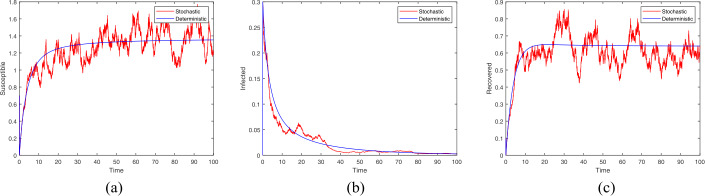
Trajectories of stochastic and deterministic systems with $R_{0}<1$

Hence, by Theorem 4.1, all positive solutions of the model (3) may fluctuate around the rumor-free equilibrium of the model (1). As clearly illustrated in Fig. 1, the curve of the stochastic model goes up and down around the curve of the deterministic model, which strongly supports Theorem 4.1. Meanwhile, we get that when $R_{0} < 1$, the number of infected individuals diminishes gradually until disappearing, which indicates the rumor can be effectively controlled.

Besides, we take the initial value $(S(0),I(0),R(0)) = (0.7,0.1,0.1)$ with related parameters: $p = 0.2$, $b = 0.6$, $\mu = 0.25$, $\beta = 0.6$, $\alpha = 0.1$, $\eta = 0.1$, $\tau = \ln 8$, $\sigma _{1} = 0.1$, $\sigma _{2} = 0.1$, $\sigma _{3} = 0.1$. In this situation, $R_{0} = 2.796 > 1$ and the conditions in Theorem 5.1 are satisfied:

$$\begin{aligned}& \mu ^{2} = 0.0625 > 0.06 = \eta b,\qquad \frac{y(\mu ^{2} - \eta b)\mu }{y(\mu ^{2} - \eta b) + 2\mu ^{2}} = 0.023 > 0.01 = \sigma _{1}^{2}, \\& \mu + x\eta = 0.337 > 0.01 = \sigma _{2}^{2},\qquad \biggl(\frac{y}{2} + 2\biggr)\mu = 1.12 > 0.01 = (y + 1)\sigma _{3}^{2}, \\& I^{*} = 0.376,\qquad R^{*} = 0.632,\qquad x = 0.865, \qquad y = 4.96. \end{aligned}$$

**Figure 2:**
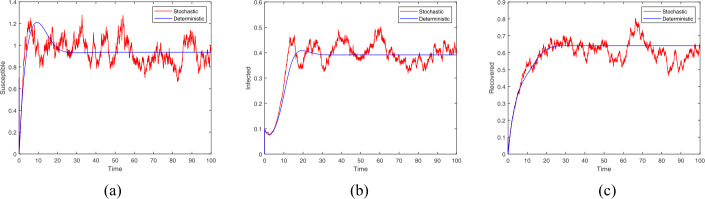
Trajectories of stochastic and deterministic systems with $R_{0}>1$

According to Theorem 5.1, all positive solutions of the model (3) fluctuate around the rumor-local equilibrium of the model (1). Similarly, the curve of the stochastic model goes up and down around the curve of the deterministic model in Fig. 2, which clearly supports this analytical result. Meanwhile, we find that when $R_{0} > 1$, the three types of group exist in the system simultaneously and ultimately each tends to stabilize. However, once influenced by external disturbance, the stable state will be broken and the rumor will keep on spreading.

**Figure 3:**
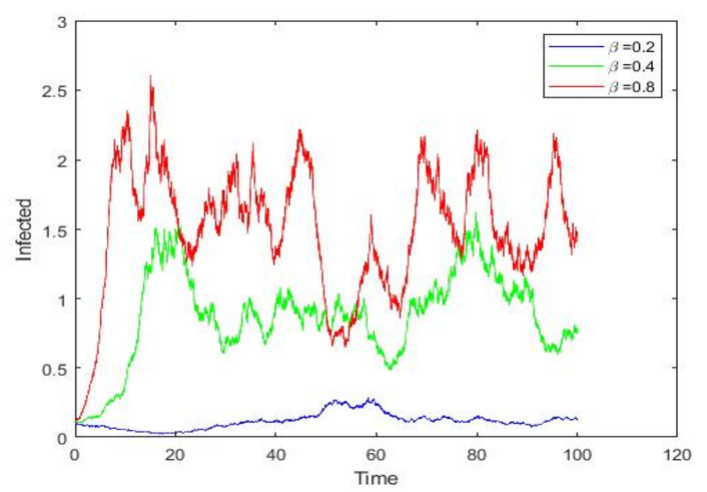
Paths simulations of $I(t)$ for stochastic model and $\beta = 0.2, 0.4, 0.8$, respectively

In addition, we are interested in what factors will influence the spread of a rumor. To begin with, the transmission rate is regarded as an indispensable component to rumor spreading. Here, under the condition that other factors are the same, we choose different transmission rate $\beta = 0.2, 0.4, 0.8$ to explore their influence on rumor spreading. As clearly shown in Fig. 3, reducing the transmission rate may decrease the number of infected individuals accordingly. For example, during the outbreak of COVID-19, when a rumor such as ‘Asymptomatic infected people are characteristic of novel coronavirus in later stages’ came to some people who haven’t heard it, if they didn’t believe it or have interest in spreading it, that the transmission rate would decrease and the spread of this rumor would be suppressed.

**Figure 4:**
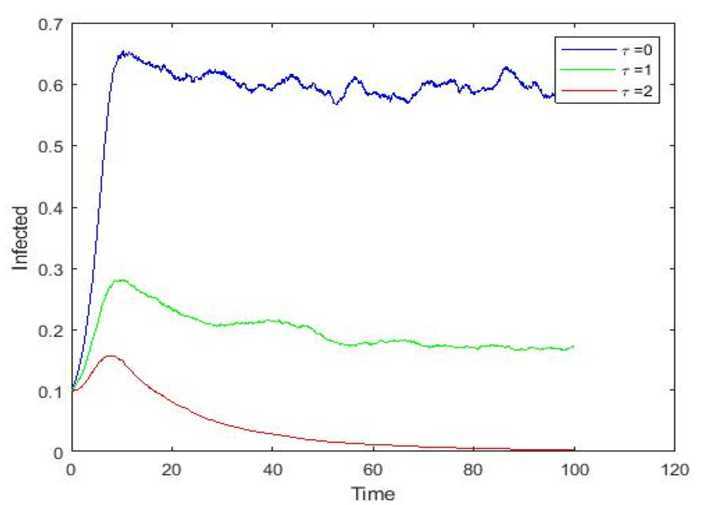
Paths simulations of $I(t)$ for stochastic model and $\tau=0,1,2$, respectively

Moreover, we consider that the time delay might exert a certain influence on rumor spreading. Thus, we change the time delay while keeping all the other parameters unchanged. As clearly shown in Fig. 4, we find that the time delay *τ* has a great influence on whether the rumor exists or not, extending the thinking time may decrease the number of the infected individuals, and it can even lead to the disappearance of a rumor. Consequently, we conclude that rumor spreading often becomes increasing fierce through those who spread automatically when hearing a rumor. As the saying goes, the rumor stops at a wise person. In this regard, we urgently need to improve our national comprehensive quality, increase our knowledge base, and improve the ability of self-prevention and control. In the face of a rumor, people should think calmly about the issue and judge truth and falsehood rationally, in which case we can control the rumor spreading effectively. Take COVID-19, for example, when groundless rumor like ‘Bee venom can inhibit a novel coronavirus’ started to spread, if individuals chose to spend some time conducting a little checking and thinking to identify instead of thoughtless spreading, the rumor would disappear naturally.

**Figure 5:**
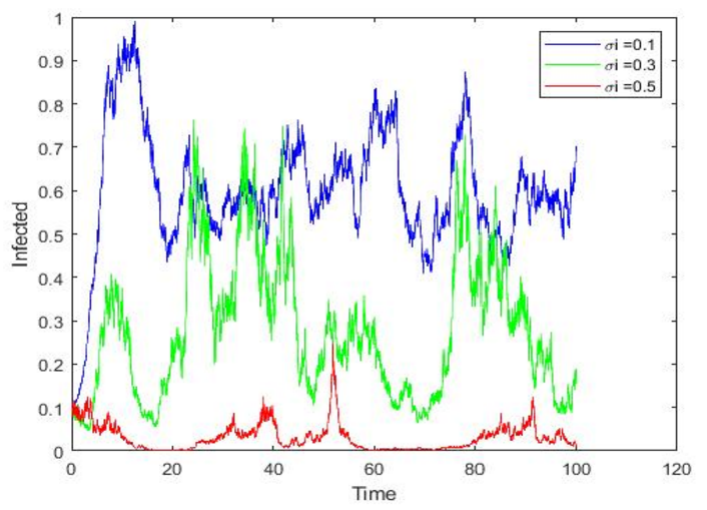
Paths simulations of $I(t)$ for stochastic model and $\sigma_{i}=0.1,0.3,0.5$, respectively

Besides what’s mentioned above, we firmly believe the scale of infected individuals must have a significant relationship with external stochastic disturbance. So under the condition that other factors are the same, we choose different intensities of white noise $\sigma_{i}=0.1,0.3,0.5$. Figure 5 demonstrates strongly that stochastic disturbance exerts far-reaching influence on the scale of infected individuals, and positive stochastic factors will reduce the scale of rumor spreading. Consequently, confronted with a rumor, the government should strengthen supervision, establish a mechanism to refute rumors, and step up suggestions how to deal with rumors. In addition, media and related platforms should pronounce the truthful, transparent, and open information to refute rumors timely. During the outbreak of COVID-19, some rumors such as ‘Hubei is short of food and people need to grab rice and oil’ and ‘Schools at all levels in Hebei are scheduled to start on April 10’, and so on, occurred, the China Internet joint rumor refutation platform released the true information to refute rumors timely, which greatly suppressed the spread of rumors.

## Conclusions

This paper is mainly related to the dynamical properties of a stochastic SIR rumor-spreading model with Holling II functional response function considering the existence of time delay and the disturbance of white noise. Firstly, we explored the existence and uniqueness of the global positive solution to the model. Besides, the asymptotic behavior of the solutions around the rumor-free and rumor-local equilibria was analyzed in detail. Furthermore, some numerical results illustrated that when reducing the transmission rate or increasing the time delay or positive external disturbance, it may suppress rumor spreading to some extent, so that the rumor vanishes. Therefore, on the one hand, we need to develop our national comprehensive literacy, expand our scope of knowledge, and improve resolving ability, then we will not believe rumors easily while spending more time thinking and identifying after contacting with spreaders, which will suppress the rumor spreading as soon as possible; on the other hand, the government and media should announce the truth in time to refute a rumor, which will avoid unnecessary panic in the crowd and guide people to act correctly.

## Data Availability

Please contact author for data requests.
